# Prepartum dietary energy intake alters adipose tissue transcriptome profiles during the periparturient period in Holstein dairy cows

**DOI:** 10.1186/s40104-019-0409-7

**Published:** 2020-01-03

**Authors:** Andrea Minuti, Massimo Bionaz, Vincenzo Lopreiato, Nicole A. Janovick, Sandra L. Rodriguez-Zas, James K. Drackley, Juan J. Loor

**Affiliations:** 1 Department of Animal Sciences,Food and Nutrition, Faculty of Agriculture, Food and Environmental Science, Università Cattolica del Sacro Cuore, 29122 Piacenza, Italy; 2 Animal and Rangeland Sciences, Oregon State University, Corvallis, OR 97330 USA; 3 Department of Animal Sciences, Division of Nutritional Sciences, University of Illinois, Urbana, IL 61801 USA

**Keywords:** Periparturient cow, Prepartum overfeeding, Subcutaneous adipose tissue, Transcriptome

## Abstract

**Background:**

The aim of the study was to investigate the effect of energy overfeeding during the dry period on adipose tissue transcriptome profiles during the periparturient period in dairy cows.

**Methods:**

Fourteen primiparous Holstein cows from a larger cohort receiving a higher-energy diet (1.62 Mcal of net energy for lactation/kg of dry matter; 15% crude protein) for ad libitum intake to supply 150% (OVR) or 100% (CTR) of energy requirements from dry off until parturition were used. After calving, all cows received the same lactation diet. Subcutaneous adipose tissue (SAT) biopsies were collected at − 14, 1, and 14 d from parturition (d) and used for transcriptome profiling using a bovine oligonucleotide microarray. Data mining of differentially expressed genes (DEG) between treatments and due to sampling time was performed using the Dynamic Impact Approach (DIA) and Ingenuity Pathway Analysis (IPA).

**Results:**

There was a strong effect of over-feeding energy on DEG with 2434 (False discovery rate-corrected *P* < 0.05) between OVR and CTR at − 14 d, and only 340 and 538 at 1 and 14 d. The most-impacted and activated pathways in the Kyoto Encyclopedia of Genes and Genomes (KEGG) database that were highlighted by DIA analysis at − 14 d in OVR vs. CTR included 9 associated with carbohydrate metabolism, with ‘Pyruvate metabolism’, ‘Glycolysis/gluconeogenesis’, and ‘Pentose phosphate pathway’ among the most-activated. Not surprisingly, OVR led to marked activation of lipid metabolism (e.g. ‘Fatty acid biosynthesis’ and ‘Glycerolipid metabolism’). Unexpected metabolic pathways that were activated at − 14 d in OVR included several related to metabolism of amino acids (e.g. branched chain) and of cofactors and vitamins (thiamin). Among endocrine and immune system pathways, at − 14 d OVR led to marked activation of ‘PPAR signalling’ and ‘Antigen processing and presentation’. Among key pathways affected over time in OVR, a number were related to translation (e.g. mTOR signaling), endocrine/immune signaling (CXCR4 and IGF1), and lipid metabolism (oxidative phosphorylation) with greater activation in OVR vs. CTR specifically at − 14 d. Although statistical differences for several pathways in OVR vs. CTR nearly disappeared at 1 and 14 vs. − 14 d, despite the well-known catabolic state of adipose depots after calving, the bioinformatics analyses suggested important roles for a number of signaling mechanisms at − 14 vs. 14 than 1 vs. -14 d. This was particularly evident in cows fed to meet predicted energy requirements during the dry period (CTR).

**Conclusions:**

Data underscored a strong activation by overfeeding energy of anabolic processes in the SAT exclusively prepartum. The study confirmed that higher-energy diets prepartum drive a transcriptional cascade of events orchestrated in part by the activation of PPARγ that regulate preadipocyte differentiation and lipid storage in SAT. Novel aspects of SAT biology to energy overfeeding or change in physiologic state also were uncovered, including the role of amino acid metabolism, mTOR signaling, and the immune system.

## Background

The transition from pregnancy into lactation is characterized by dramatic and sudden physiological changes, and it is recognized to be the most difficult stage in the dairy cow’s life [1, 2]. During this period, metabolic adaptations in major organs (e.g., mammary, liver, rumen, and adipose) are coordinated to allow the animal to satisfy the needs for synthesizing milk. Although the concerted biological roles of liver and mammary during the transition period in coordination of animal physiology are well known [3], similar knowledge for adipose tissue activity is scant [4].

Adipose tissue is not simply a metabolic tissue that primarily participates in regulating whole body energy homeostasis, it also plays an important endocrine function (at least in non-ruminants) by secreting a number of proteins with signaling properties that are involved in the regulation of metabolism (adiponectin, leptin), feed intake (leptin), and immune function and inflammation [5, 6]. Despite the dominance of mature adipocytes, adipose tissue is also composed of immune cells (macrophages) and stromal-vascular cell fractions containing pre-adipocytes, endothelial cells, and mesenchymal stem cells, which may vary in their response to external stimuli (such as nutrient supply) and immune activation [5].

Recent work aimed at investigating transcriptomic adaptations of adipose tissue during the transition period revealed several changes in expression of genes involved in the regulation of lipid metabolism in particular, but also immune-related functions [4, 7, 8]. Among the factors that can modulate the success during the transition period, energy intake is one of the most-studied [9–11]. In that context, adipose tissue appears to be very sensitive to energy status of the organism [6]. Energy overnutrition in the dry period and the resulting over conditioning of periparturient dairy cows [12] represent possible risk factors for optimal health status around calving.

Previous experiments reported that energy overfeeding in the prepartum is often associated with negative effects in postpartum health indices, underscoring possible detrimental effects of this nutritional approach [13, 14]. In particular, the negative effect of prepartum overfeeding causes greater postpartum adipose tissue mobilization, increased risks of ketosis and fatty liver postpartum [15–17]. Therefore, the objective of the present research was to study the effect of energy overnutrition during the dry period on the adipose tissue transcriptome during the transition period. A subset of cows from the study of Janovick and Drackley [12] were used for transcriptome profiling.

## Materials and methods

### Animal management

All procedures were conducted under protocols approved by the University of Illinois Institutional Animal Care and Use Committee. The experimental design, management details and details of ingredients and nutrient composition of diets have been published previously [12, 15]. Briefly, 14 cows (7 per treatment group) from the larger cohort entering their first lactation were randomly selected for adipose tissue biopsy. All cows were moved to individual ties stalls at 65 d before expected parturition and were fed the herd dry cow diet. From 42 d before expected parturition to parturition, cows were assigned to a high-energy diet fed for ad libitum intake (OVR) to provide at least 150% of NRC energy requirements for dry cows in late gestation, or to a control-energy diet (CTR) to limit energy intake to 100% of NRC requirements at ad libitum intake [18]. The latter was achieved using chopped wheat straw at 31.8% of the DM (Table 1). Initial average body condition score was 3.58 and 3.50 (5-point scale) and body weight 569 and 592 kg for CTR and OVR. Cows remained on their respective treatments until parturition. The same lactation diet was fed to all cows during lactation (Table 1).

**Table 1:** Ingredients and nutrient composition of diets fed to during prepartum and early lactation

Item	Prepartum^a^		Lactation
	CTR	OVR	
Ingredient, % of DM			
Corn silage	35.5	35.8	30.5
Alfalfa silage		13.3	18.6
Alfalfa hay	17.2	9.5	2.0
Wheat straw, chopped	31.8		
Cottonseed		5.1	9.5
Corn grain, ground	3.6	17.9	20.7
Soybean meal, 48% CP	5.1	6.6	6.0
Soybean meal, expeller	4.1		4.0
Soy hulls		10.4	3.0
Wheat middlings			3.0
Vitamin and mineral mix^b^	0.3	0.3	0.3
Salt	0.3	0.2	0.2
Dicalcium phosphate	0.1	0.1	0.1
Sodium bicarbonate			0.9
Limestone	0.8		1.0
Magnesium oxide			0.1
Magnesium sulfate	0.3	0.2	0.1
Vitamin A^c^	0.1	0.1	0.1
Vitamin D^d^	0.1	0.1	0.1
Vitamin E^e^	0.2	0.1	0.1
Urea	0.9	0.2	
Nutrient content^f^			
% DM	54.1	53.2	53.3
CP, % of DM	14.2	15.0	17.1
ADF, % of DM	24.7	26.5	23.0
NDF, % of DM	51.9	38.6	35.4
NE_L_, Mcal/kg of DM	1.21	1.63	1.69

^a^Diets were fed to cows from −42 d relative to expected parturition to parturition

^b^Contained a minimum of 5.0% Mg, 10.0% S, 7.5% K, 2.0% Fe, 3.0% Zn, 3.0% Mn, 5,000 mg/kg Cu, 250 mg/kg I, 40 mg/kg Co, 150 mg/kg Se, 2,200,000 IU/kg of vitamin A, 660,000 IU/kg of vitamin D_3_, and 22,000 IU/kg of vitamin E

^c^Contained 30,000 kIU/kg

^d^Contained 5,009 kIU/kg

^e^Contained 44,000 IU/kg

^f^Nutrient composition based on 4-week feed ingredient composites

### Adipose tissue biopsy and handling

Subcutaneous adipose tissue biopsies were collected from alternate sides of the tail-head region at − 14, 1, and 14 d from parturition, before the morning feeding. The hair of the surgical area was cut closely with clippers and washed with an iodine disinfectant mixture. Lidocaine-HCl (5 mL; Agri Laboratories) was given intramuscularly to anaesthetize the biopsy area 10 min before performing a ~ 2-cm incision. Adipose tissue (2–4 g) was collected with scalpel and forceps by blunt dissection. The incision was then closed with surgical staples (Multi-Shot Disposable Skin Stapler, Henry Schein) and iodine ointment was applied to the wound. The wound was carefully monitored for the following 7 d. The tissue was quickly blotted with sterile gauze to remove residual blood and snap-frozen in liquid N until RNA extraction for gene expression analysis.

### RNA extraction and microarrays analysis

Procedures for RNA extraction and microarray analysis have been described previously [19]. The transcript profiling was done using a bovine oligonucleotide (70-mer) microarray with > 13,000 annotated sequences developed at the University of Illinois [19]. Details of the development, annotation, use of this microarray, and methods for microarray hybridization and scanning have been reported previously [19].

### Statistical analyses

Microarray spots with median intensity ≥3 standard deviation above the median of the background and GenePix 6 flag > 100 were applied as filters to ensure high quality data. A total of 106 microarrays were adjusted for dye and array effect (Loess normalization and array centering), duplicated spot intensities were not averaged and were subsequently used for statistical analysis. A mixed model with repeated measures was then fitted to the normalized log_2_-transformed adjusted ratios (sample/reference standard) using Proc MIXED (SAS, SAS Inst. Inc., Cary, NC). The model included the fixed effects of time (− 14, 1, 14 d), diet (OVR and CTR), and interactions of time × diet. Cow was considered as a random effect. The *P*-values were adjusted for the number of genes tested using Benjamini and Hochberg’s false discovery rate (FDR) to account for multiple comparisons [20]. Differences in relative gene expression were considered significant at an FDR-adjusted *P* ≤ 0.05 for time × diet. A *P* ≤ 0.05 was considered significant between diets at each time point.

### Ingenuity pathway analysis

Ingenuity Pathway Analysis (IPA, Ingenuity Systems) was used to identify enriched pathways in each comparison. The whole annotated dataset with FDR, expression ratio, and *P*-value for each comparison was uploaded in IPA. The whole annotated dataset was used as background and the default databases in IPA were used. IPA was also used to identify up-stream regulators as described previously [21]. Network analysis was also performed using IPA.

### Dynamic impact approach

The Dynamic Impact Approach (DIA) was used to identify impact and direction of the impact in KEGG pathways determined by DEG as previously described [17]. Only pathways with at least 4 genes present in the annotated microarray were considered.

## Results

The number of differentially expressed genes (DEG) according to up-regulation or down-regulation are displayed in Table 2. The complete dataset is available in Additional file 1. The transcriptome during the transition from pregnancy to lactation was more affected in OVR compared with CTR. We detected a large number of DEG between OVR and CTR at − 14 d with a total of 2434 DEG. After calving, the number of DEG between OVR and CTR was lower with only 340 and 538 DEG at 1 and 14 d, respectively.

**Table 2:** Differentially expressed genes (False discovery rate *P* < 0.05) in adipose tissue from dairy cows fed a control diet (CTR) or a higher-energy diet prepartum (OVR) across different time point comparisons during late pregnancy and early lactation (− 14 d, 1 and 14 d)

Comparison	DEG	UP	DOWN
CTR			
1 vs. −14	600	320	280
14 vs. −14	1176	681	495
14 vs. 1	986	510	476
OVR			
1 vs. −14	2919	1482	1437
14 vs. −14	1612	827	785
14 vs. 1	2427	1160	1267
CTR vs. OVR			
−14	2434	1240	1194
1	340	168	172
14	538	298	240

### Impact of transition into lactation

**Fig. 1:**
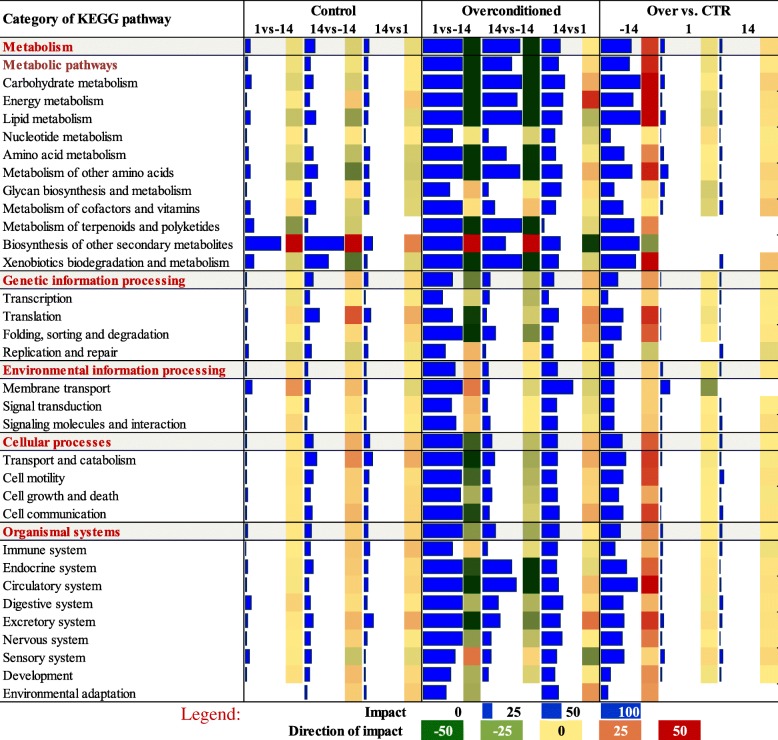
Summary of transcriptomic effects on KEGG pathways in adipose tissue from dairy cows fed a control diet (CTR) or a higher-energy diet prepartum (OVR). Data encompass the end of pregnancy (− 14 d) through early lactation (1 and 14 d). The data were analyzed using the Dynamic impact Approach (DIA). Shown are the impact values (blue horizontal bars) and the direction of the impact values (red shade denotes activation and green denotes inhibition)

**Fig. 2:**
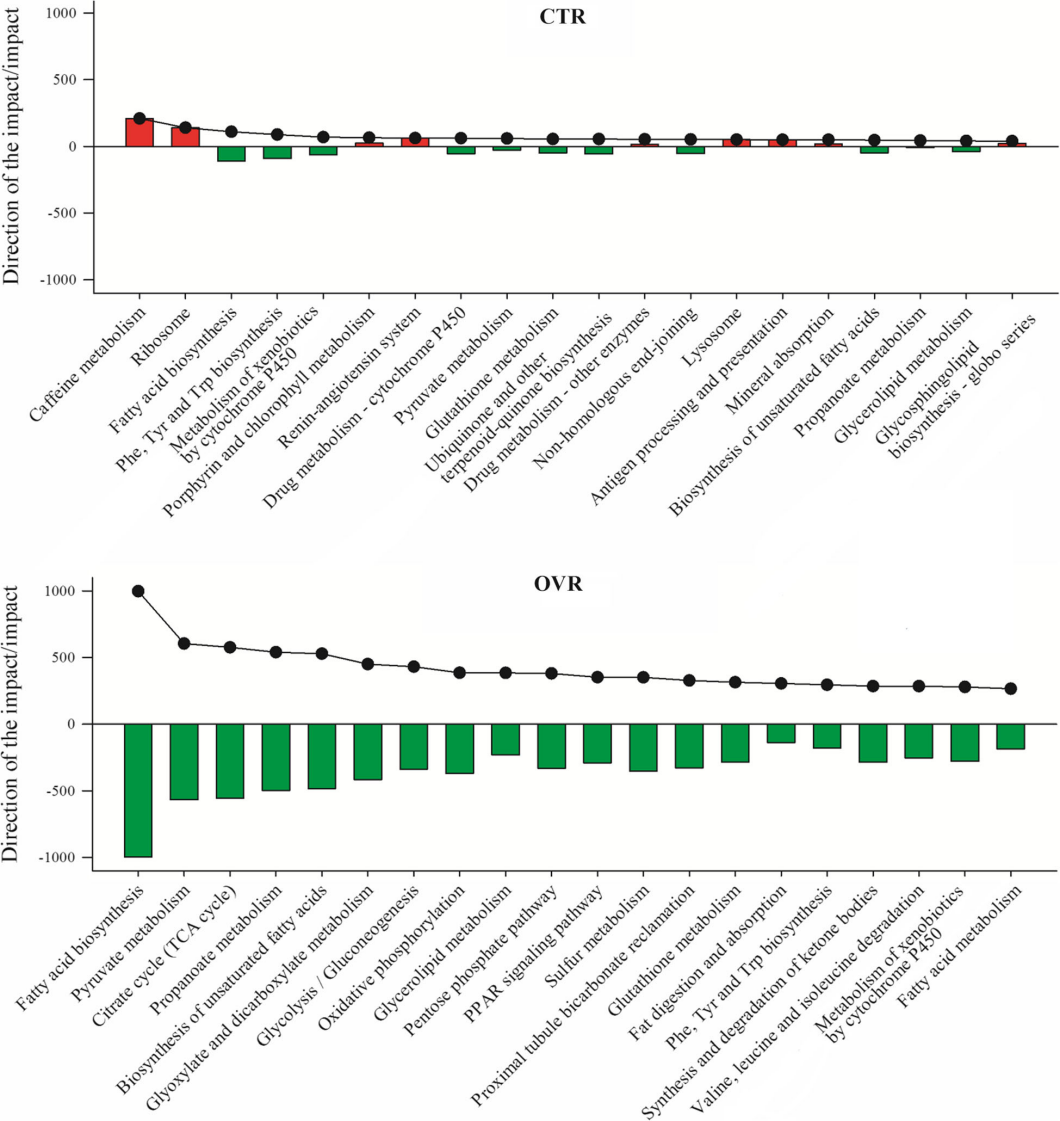
Metabolic adaptations of adipose tissue from end of pregnancy through early lactation (1 and 14 d) in dairy cows fed a control diet (CTR) or a higher-energy diet prepartum (OVR). Shown are outputs (i.e., direction of the impact and impact) of selected KEGG pathways from bioinformatics analysis using the Dynamic Impact Approach (DIA) and differentially expressed genes at 1 and 14 d post-partum compared with − 14 d pre-partum. The panels depict the impact (black line and dots) and the direction of the impact (bars; positive red bars denote activation while negative green bars inhibition) for the most impacted pathways in the KEGG subcategories

**Fig. 3:**
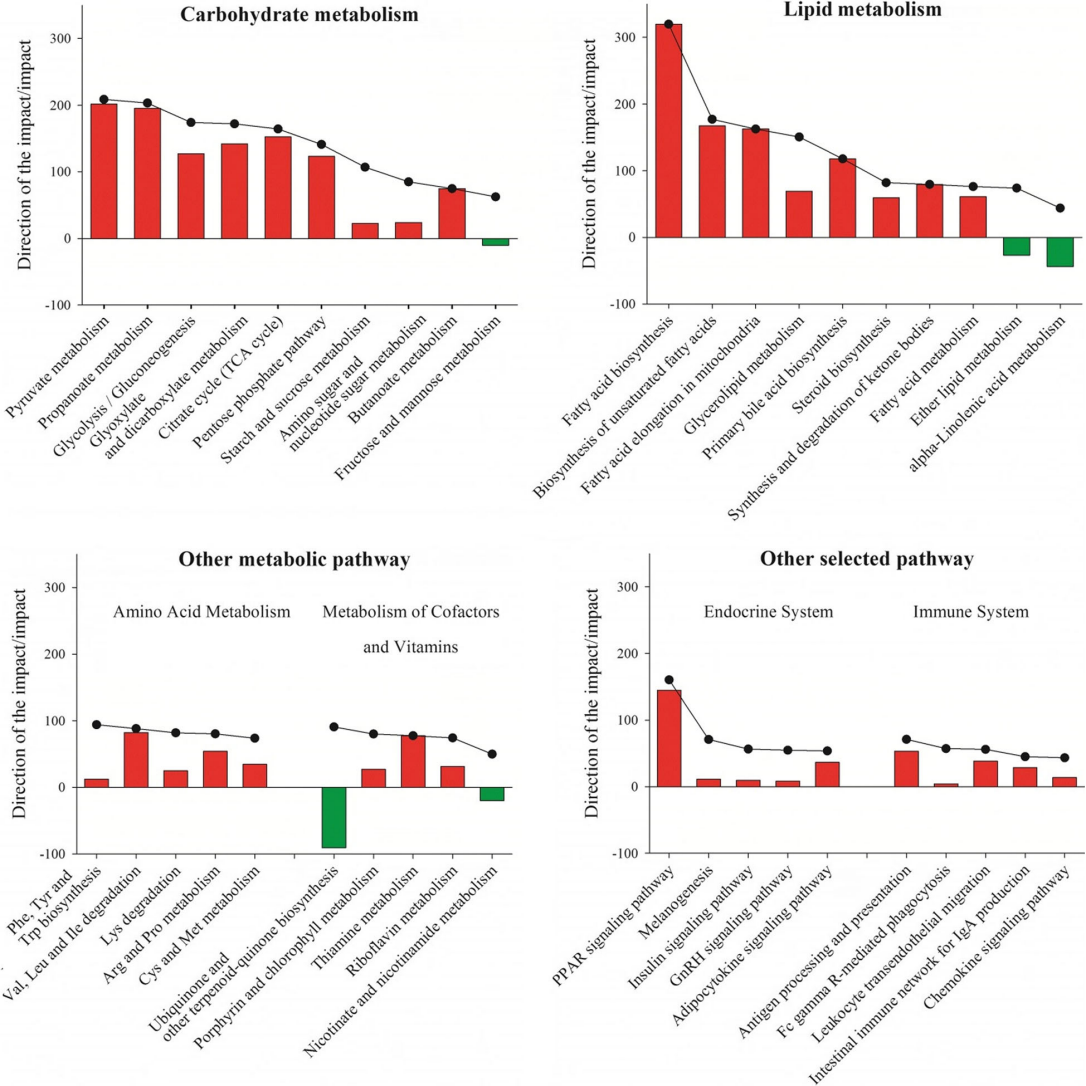
Metabolic differences of adipose tissue at the end of pregnancy at − 14 d in dairy cows fed a control diet (CTR) or a higher-energy diet prepartum (OVR). Shown are outputs (i.e., direction of the impact and impact) of selected KEGG pathways from bioinformatics analysis using the Dynamic Impact Approach (DIA) and differentially expressed genes at − 14 d pre-partum for the most impacted pathways in the KEGG subcategory ‘Lipid metabolism’, ‘Carbohydrate metabolism’, ‘Amino acid metabolism’, and other selected pathways. The panels depict the impact (black line and dots) and the direction of the impact (bars; positive red bars denote activation while negative green bars inhibition) for the most impacted pathways in the KEGG subcategories

Figure 1 depicts the pathway summary generated by DIA analysis for the impact value and the direction of the impact for the major KEGG pathway categories. Details are available for all the pathways in Additional file 2. Figures 2 and 3 depict the direction of impact for the most impacted pathways. There was a lesser impact of change in stage of lactation in CTR compared with OVR cows. Pathways related to metabolism were the most impacted in all comparisons.

Among metabolic-related pathways, the most impacted in the CTR group were ‘Biosynthesis of other secondary metabolites’ driven by ‘Caffeine metabolism’, which was activated after calving, and ‘Xenobiotics biodegradation and metabolism’ driven by P450 enzyme-related pathways that was inhibited during lactation (Fig. 1). With a minor impact in CTR among categories of pathways, we detected an activation of ‘Translation’ and an inhibition of ‘Metabolism of other amino acids’, especially ‘Phe, Tyr and Trp biosynthesis’.

In OVR cows, the DIA analysis revealed a large number of highly impacted categories of KEGG pathways during the transition from the dry period to lactation; especially at 1 vs. −14 d (Fig. 1). There was an overall inhibition of all pathways during the transition into lactation. Among categories of pathways, the most inhibited were related to metabolism including carbohydrate and lipid. Especially inhibited were pathways related to synthesis of triglycerides via use of glucose, such as pyruvate metabolism and TCA cycle, with ‘Fatty acid biosynthesis’ as the top-affected pathway. The second most inhibited category of pathways was ‘Cellular processes’, where pathways related to catabolism and cell proliferation were the most-affected (Fig. 1 and Additional file 2). A marked inhibition of metabolism and cell proliferation in OVR was detected only during the last 2 weeks of pregnancy while during the first 2 weeks of lactation (i.e., 14 vs. 1 d) (with the exception of “Biosynthesis of Other Secondary Metabolites”) most of the same pathways were induced. Besides metabolic-related pathways, also translation, protein degradation, transport and catabolism, cellular communication, and endocrine systems were strongly inhibited. In the latter categories of pathways, the most affected was ‘PPAR signaling pathway’. This pathway was slightly affected in CTR cows, but was among the most different between the two groups at − 14 d and one of the most inhibited in OVR from − 14 to 1 d, together with pathways related to triglyceride synthesis (Fig. 2 and Additional file 2).

Most affected pathways between CTR and OVR at − 14 d are reported in Fig. 3. Compared with CTR, before calving the OVR group had higher activation of pathways involved in carbohydrate metabolism including ‘Pyruvate metabolism’, ‘Propanoate metabolism’, ‘Glycolysis/gluconeogenesis’, ‘Citrate cycle (TCA cycle)’, and ‘Pentose phosphate pathway’. At the same time, compared with CTR, pathways related to lipid metabolism had a higher degree of activation (Fig. 3). Among the most activated pathways were those related to triglyceride synthesis including ‘Fatty acid biosynthesis’, ‘Biosynthesis of unsaturated fatty acids’, and ‘Glycerolipid metabolism’ (Fig. 3). In addition, prior to parturition, amino acid-related pathways were more activated in OVR compared with CTR; those include ‘Glutathione metabolism’, ‘Valine, leucine and isoleucine degradation’, ‘Arginine and proline metabolism’, and ‘Thiamine metabolism’. Besides metabolic-related pathways, also translation (i.e., ‘Ribosome’), especially including proteins formed in the ER, ‘Antigen processing and presentation’ among immune-related pathways, and ‘PPAR signaling’ were more activated in OVR compared with CTR (Fig. 3 and Additional file 2).

### Ingenuity pathway analysis of enriched pathways

**Fig. 4:**
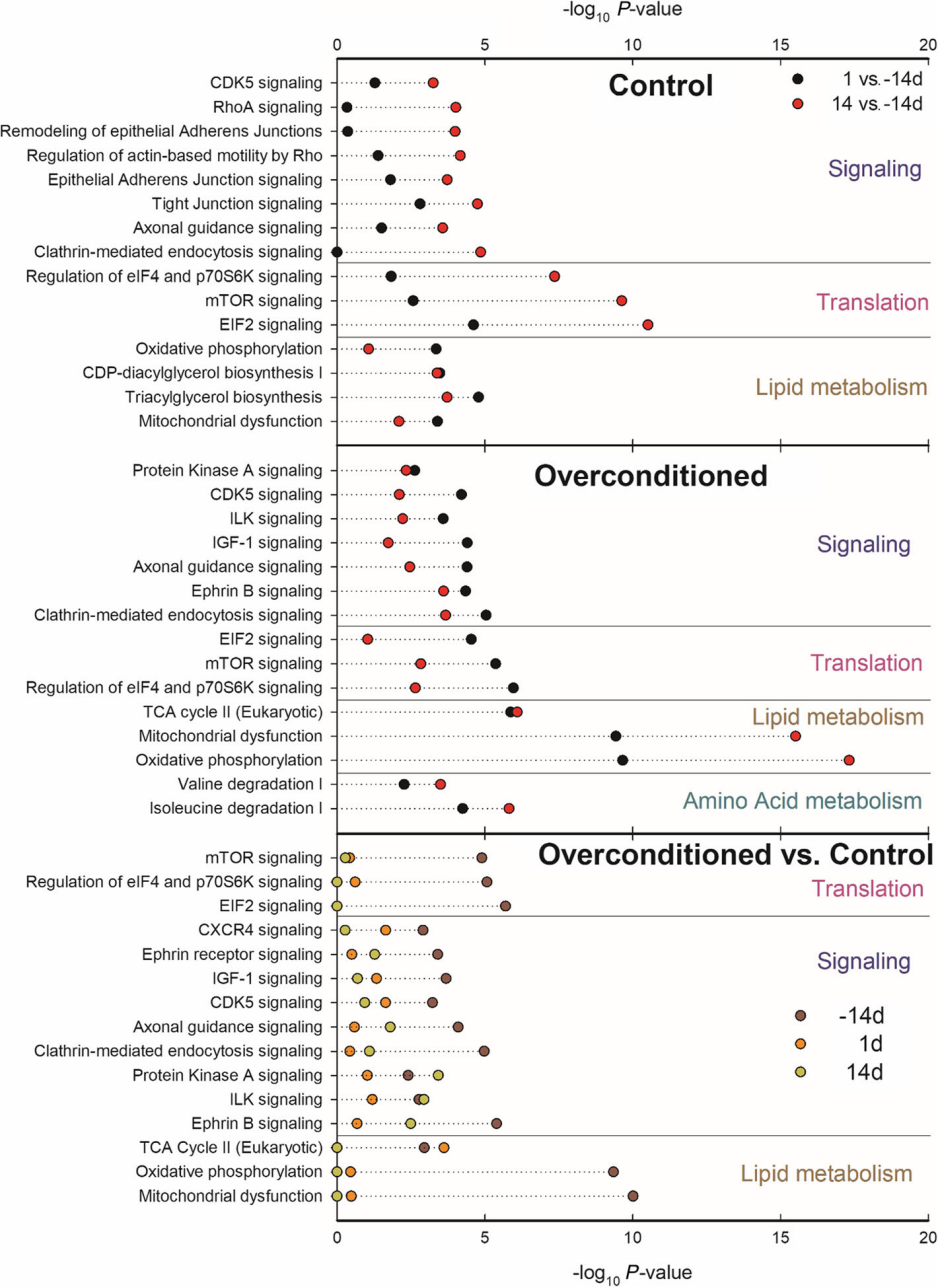
Most enriched pathways detected by Ingenuity Pathway Analysis in each comparison (≥ 1.3 –log_10_ *P*-value in at the least one comparison). Shown are the 3 –log_10_ *P*-value of enrichment and pathways grouped according to main functional clusters

The analysis using Ingenuity Pathway Analysis confirmed the importance of translation and lipid metabolism in adipose tissue during the transition from pregnancy to lactation (Fig. 4 and Additional file 3). In both CTR and OVR, the DEG affected by time were highly enriched with genes related to signaling pathways, translation, and lipid metabolism. In addition, genes with different expression between OVR and CTR were highly enriched with pathways related to signaling, including the mTOR pathway. Pathways related to lipid metabolism were also enriched; however, enriched were pathways involved in energy production using lipids.

### Ingenuity pathway analysis of transcriptional regulators

**Fig. 5:**
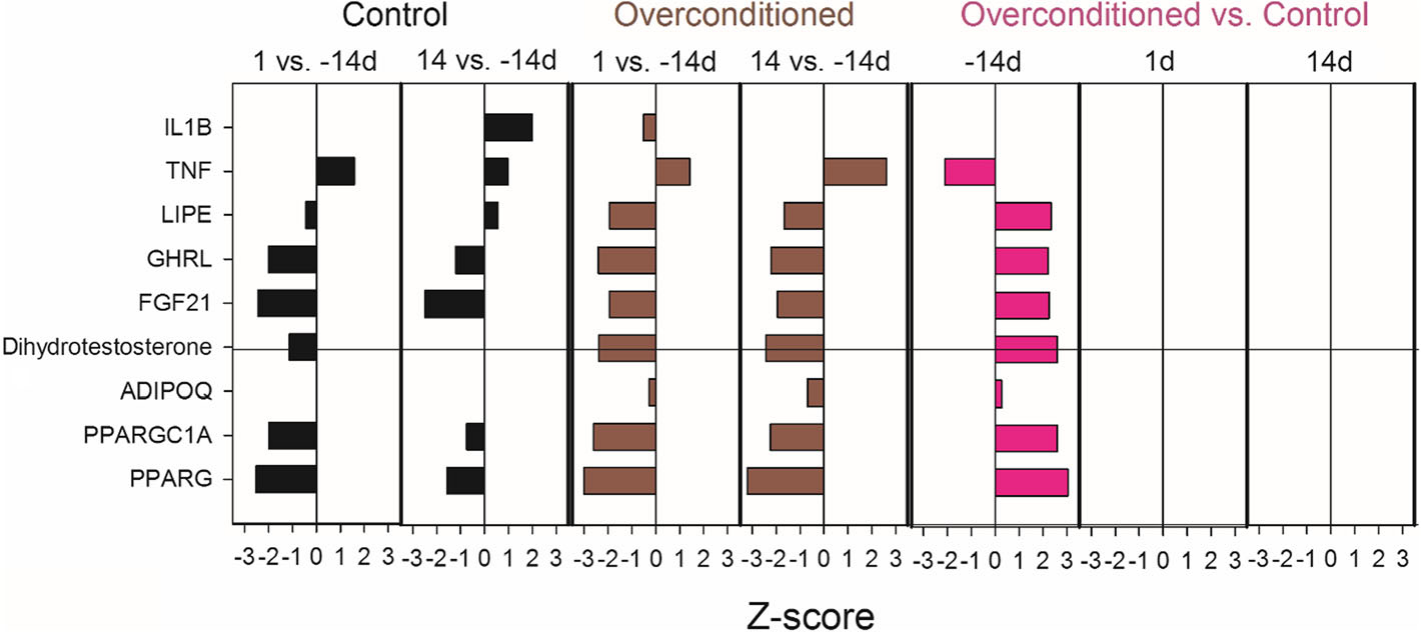
Upstream regulators estimated to be most important (absolute Z-score ≥ 2) in regulating the transcriptomic adaptations of adipose tissue in a given treatment or time comparison

**Fig. 6:**
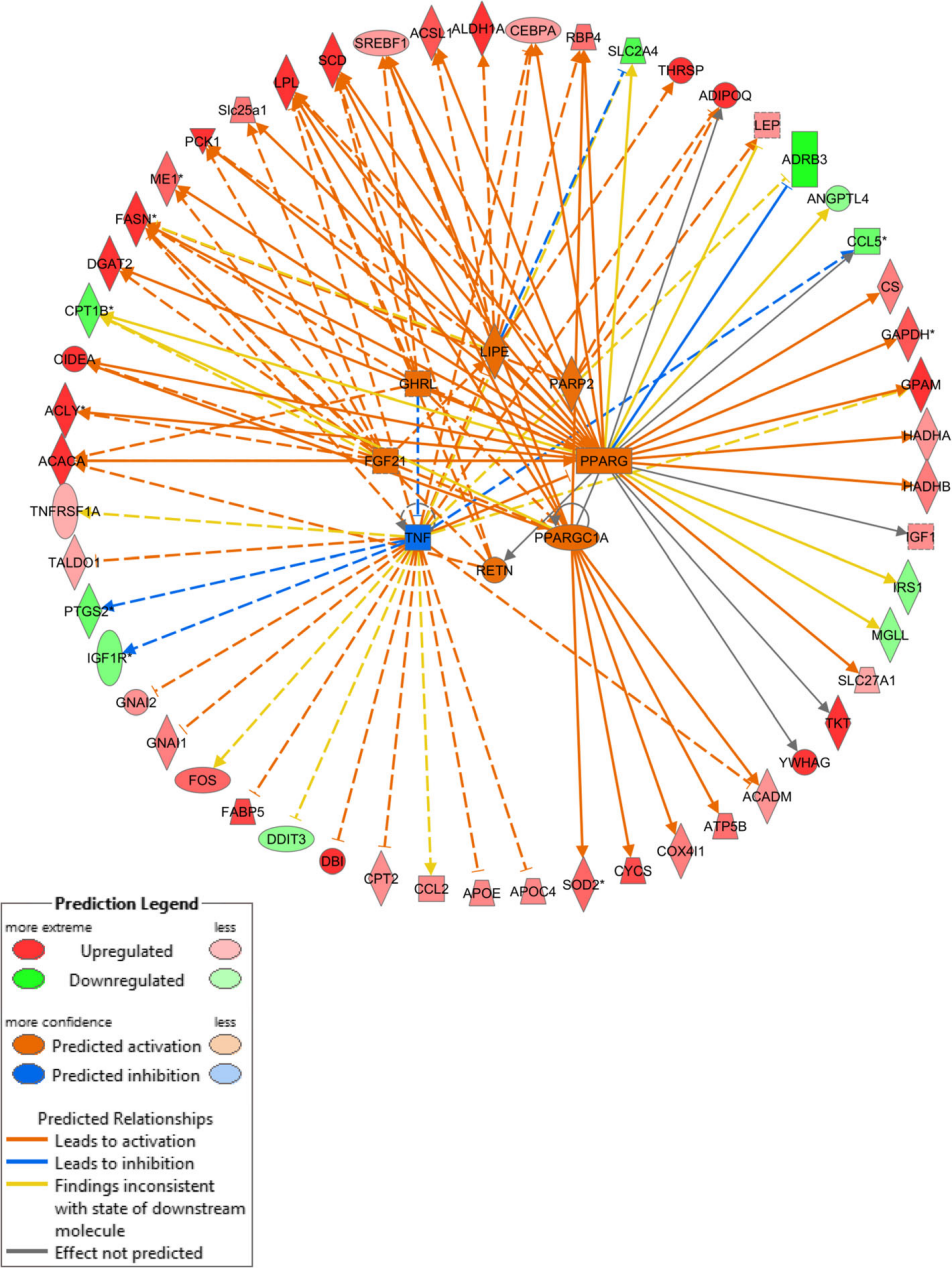
Network of up-regulators (center in the network) with the highest impact in controlling the difference in the adipose tissue transcriptome from dairy cows fed a control diet (CTR) or a higher-energy diet prepartum (OVR) at − 14 d relative to parturition. Orange shades denote activation and blue shades inhibition of the up-regulators. Red shades denote up-regulation while green shades denote down-regulation. Blue and orange dotted lines in arrows denote the inhibition and activation effect, respectively, of the up-stream regulators on target genes

The analysis using Ingenuity Pathway Analysis revealed few upstream regulators deemed important in regulating the transcription of DEG detected in this study with an estimated Z-score ≥ 2 (i.e., activated) or ≤ − 2 (i.e., inhibited) (Fig. 5). In particular, *TNF* was estimated to be inhibited in adipose tissue of OVR compared with CTR cows in the pre-partum at − 14 d (Fig. 5). Genes related to the inflammatory cascade, *CCL5* (involved in immunoregulatory and inflammatory process), and *PTGS2* (key enzyme in prostaglandin biosynthesis) were downregulated by the inhibition of *TNF* (Fig. 6)*.* Furthermore, the inhibition of *TNF* led to the upregulation of genes related to fatty acid synthesis, *ACACA* (catalyzing the carboxylation of acetyl-CoA to malonyl-CoA, the rate-limiting step in fatty acid synthesis) and *FASN* (which catalyze the synthesis of long-chain saturated fatty acids). The latter was the most upregulated gene (Fig. 6).

Except for cytokines such as TNF and IL1B, all upstream regulators in both CTR and OVR were estimated to be inhibited in adipose tissue during the transition from pregnancy to lactation. The most important inhibited upstream molecules were the nuclear receptor PPARγ and the growth factor FGF21 and ghrelin (GHRL). These also were among the most induced in OVR and CTR animals at − 14 d. These upstream regulators form a tight gene network (Fig. 6). It is noteworthy to highlight that in the network displayed in Fig. 6, several genes related to insulin signaling (i.e., *IRS1*) and glucose uptake (i.e., *SLC2A4*) were down-regulated, in OVR vs. CTR at − 14 d despite being PPARγ target genes.

## Discussion

Few studies have evaluated changes in adipose tissue during the transition period, but they have revealed a dramatic transcriptome downregulation after parturition with a quick interruption of many anabolic functions related to lipogenesis [4, 22]. On the basis of gene expression data generated by RT-qPCR, overfeeding high yielding dairy cows in the prepartal period resulted in slight alterations of the transcriptional adaptations of crucial genes in subcutaneous adipose tissue and did not compromise the insulin signaling pathway [7, 16]. However, the negative consequences of overfeeding energy during the dry period are manifested at the beginning of the new lactation when the energy stored previously as fat is mobilized.

In the present study, the subcutaneous adipose tissue was more affected by energy content of the diet than the physiological change at the onset of lactation. This was evidenced by the relatively low number of DEG during the transition in cows fed as recommended by the NRC [18], while cows fed a higher energy diet prepartum (150% of requirement) had almost 3000 DEG during the transition into lactation. Furthermore, despite all the cows receiving the same diet in the postpartum, the two groups had more than 500 genes with a different expression and the number of DEG was persistently greater in OVR vs. CTR cows also in the postpartum. These data underscore a carryover effect of the higher energy diet on the adipose tissue transcriptome. The effects on the transcriptome detected in our study partly support prior observations, and in particular a large and persistent effect of energy intake on lipogenesis [23–25].

The bioinformatics analysis of our data clearly indicated a strong lipogenic effect of the higher energy diet in the prepartum. This confirms that lipogenesis is mainly regulated at a transcriptional level (as in non-ruminants), while lipolysis, which is predominant during negative energy balance is mainly controlled through other mechanisms, e.g., post-translational activation by protein phosphorylation [24]. After the beginning of lactation all cows received the same diet and differences between OVR and CTR were strongly diminished, suggesting a relatively quick homeorhetic adaptation to dietary energy level, allowing its partitioning at the onset of lactation.

The diet received by the OVR cows in our experiment contained a higher amount of fermentable carbohydrate (corn grain: 17.9% in OVR vs. 3.6% in CTR). This would stimulate the production and absorption of ruminal VFA [26] and increase glucose availability [27]; hence, increasing the supply of energy that in turn results in the stimulation of lipogenesis [28]. The principal effect of changing from a conventional diet to a higher-energy diet is that propionic acid production and net energy from total VFA increase [26]. Overall, the greater biosynthesis of fatty acids from increased VFA uptake by adipose tissue of OVR compared with CTR cows is supported by the larger induction of the ‘Fatty acid biosynthesis’ pathway. Other data supporting a positive effect on lipogenesis by OVR is the strong upregulation of acetyl-CoA synthetase (*ACSS*; 7.4-fold higher in OVR vs. CTR at − 14 d) the enzyme catalyzing the synthesis of acetyl-CoA from acetate. We speculate that through changes in that enzyme, large amounts of acetate were absorbed from adipose tissue and used for de novo fatty acid synthesis [29, 30]. The higher synthesis of fatty acids is also supported by the greater induction in OVR vs. CTR of pathways related to utilization of glucose, including the pentose phosphate pathway responsible for the production of NADPH, and production of pyruvate that enters the TCA cycle producing intermediates such as citrate [31]. The latter can produce acetyl-CoA via catalysis of ATP citrate lyase (ACLY) allowing glucose and, to a lesser extent, lactate carbon to serve as substrates for fatty acid synthesis.

The greater mRNA abundance of lipogenic genes by energy overfeeding in the present study might have been induced by a combination of greater substrate supply and insulin, which is a well-known lipogenic hormone [32]. The inclusion of ground corn in OVR compared with chopped wheat straw in the CTR diet [16] potentially provided greater amounts of substrates (acetate from ruminal fermentation and glucose derived mostly from gluconeogenesis from propionate) for lipogenesis. Despite the lack of difference in blood glucose concentration prepartum between treatments [15], the prepartum serum insulin concentration was greater and NEFA lower in OVR compared with CTR [15] confirming a greater anabolic status as previously discussed by Piccioli-Cappelli et al. [30].

The overall results described suggest a model where the greater energy intake before parturition drives a transcriptional cascade regulating pre-adipocyte differentiation (adipogenesis) and adipocyte function mainly related to energy storage. Hundreds of genes including enzymes and transcription factors coordinate the expression of proteins responsible for establishing the mature adipocyte. The central event in this network is the activation of CCAAT enhancer binding proteins (CEBP) and PPARγ, which are essential transcription regulators for the entire process [33–35]. In particular, PPARγ, highly expressed in bovine adipose tissue [36], is considered the master regulator of adipogenesis [3] and appears to control lipogenesis in response to energy level in the diet [16, 37, 38]. In non-ruminants, PPARγ controls the induction of C/EBPα [39], whereas the expression of C/EBPα appears to be required for maintaining expression of PPARγ in the mature fat cell [40]. Besides lipogenesis, PPARγ might also play a role in fatty acid oxidation [41] controlling the expression of carnitine palmitoyl transferase 2 (*CPT2*; upregulated 1.7-fold in OVR vs. CTR cows in the present study), a protein involved in the entry of long chain fatty acids into the mitochondria prior to their oxidation. Despite a greater induction of lipogenesis in OVR vs. CTR at − 14 d, our data also indicated a larger induction of fatty acid metabolism, including catabolism.

A noteworthy aspect related to PPARγ is its impact on insulin resistance. The treatment with PPARγ agonists is a clinical approach used to treat insulin resistance. Insulin insensitivity in peripheral tissues during the transition period is an important homeorhetic adaptation [42]. In our study, the increased expression of *PPARG* and the consequent effect on several target genes in OVR cows compared with CTR may be considered a homeorhetic mechanism that acts to balance the otherwise greater insulin resistance in overfed dairy cows [16]. We previously proposed that the increased abundance and activation pre-partum of *PPARG* in adipose tissue can help alleviate the large NEFA surge due partly to the control it exerts on transcription of the insulin-sensitive glucose transporter (*SLC2A4*) plus other genes favoring lipogenesis and esterification (e.g. *FASN*, *PCK1*). This would lead to reduced lipid overload on the liver with a consequent decrease in susceptibility to lipidosis and other potential detrimental effects on metabolic health [36].

One of the most-novel outcomes from the present study was the discovery of alterations in metabolic pathways related to amino acid metabolism in OVR cows. For instance, we observed an activation of branched-chain amino acid (BCAA) catabolism, the increase of which (at least in non-ruminants) is coordinated by PPARγ and is essential to support adipocyte differentiation and lipogenesis [43, 44]. In addition to their role as indispensable components for life, the BCAA valine, leucine, and isoleucine (most-abundant of the circulating essential amino acids) are key regulators of protein synthesis, protein degradation, and insulin secretion and synthesis [45]. In mammals, the BCAA are initially transaminated by branched chain amino transferases (BCAT) to form branched chain α-ketoacids (BCKA), representing the first step of BCAA catabolism. The last step of BCAA catabolism (all reactions occur within the mitochondrial matrix) provide carbon skeletons that are either lost as CO_2_ or enter the TCA cycle. Furthermore, BCAA metabolism contributes to the synthesis of several lipid species including branched chain fatty acids, odd-chain fatty acids, and *N*-acyl amino acids. Indeed, adipocytes (particularly) can synthesize odd-chain fatty acids by combining propionyl-CoA (carbon derived from valine and isoleucine) and malonyl-CoA, followed by fatty chain elongation via fatty acid synthase [43, 46].

Although there are no published data in ruminants to help understand the specific connection between activation of BCAA degradation and activation of lipid metabolism-related pathways as direct consequence of energy overfeeding, there are some *in vitro* studies for such a connection. Crown et al. [46] using cultured 3 T3-L1 adipocytes where the medium was supplemented with radiolabeled valine, leucine and isoleucine demonstrated that at least 25% of lipogenic acetyl-CoA was derived from BCAA catabolism (leucine and isoleucine). Similarly, propionyl-CoA, precursor for odd chain fatty acids, was derived solely from isoleucine and valine (accounting for 100%), confirming the relevant contribution of BCAA to lipogenesis in differentiated adipocytes.

The activation of the ‘Valine, leucine and isoleucine degradation’ pathway implies there was greater availability of these BCAA from dietary uptake or ruminal microbiota synthesis. Compared with CTR, the greater dietary energy level in OVR cows likely improved the efficiency of microbial N synthesis. Indeed, microbial N flow to the duodenum is improved at low dietary CP content when changing from higher-fiber to higher-starch diets [47, 48]. Thus, enriching diets with highly-fermentable grain (current study: 17.9% in OVR vs. 3.6% in CTR) in the prepartum period (normally characterized by low CP and higher fiber content) leads to greater microbial N flow to the duodenum and posthepatic availability of AA [47]. In addition, the present study highlights that BCAA catabolism together with other AA-related pathways were inactivated at both 1 and 14 d compared with − 14 d. We speculate that this pattern changed following parturition likely due to the inactivation of lipid metabolism (such as fatty acid biosynthesis and PPAR signaling pathway), pyruvate metabolism, and TCA cycle. As such, the requirements of lipogenic intermediates (i.e. acetyl-CoA and propionyl-CoA) decreased, but also there was a shift in post-hepatic AA utilization from adipose tissue (prepartum) to mammary gland for milk production (postpartum). Recent data underscored that protein abundance of branched chain ketoacid dehydrogenase kinase (BCKDK) did not change between pre and postpartum periods in adipose tissue of periparturient Holstein cows, suggesting it is a response that helps channel circulating BCAA to the mammary gland [49].

In non-ruminants, adipose tissue also acts as endocrine organ and cross talks with other tissues by secretion of molecules including cytokines [50]. In particular, the adipose tissue synthesizes and secretes circulating hormones and adipokines that act as systemic inflammatory mediators and signals of the organism’s nutritional status [6]. Although transcriptional regulation of adipose by energy availability is well known in non-ruminants, such regulation in cow adipose tissue remains poorly understood. In this respect, with the application of bioinformatics analysis of the transcriptome data from adipose tissue of Holstein cows, Moisá et al. [51] shed light on changes of mRNA expression profiles of both mesenteric and subcutaneous adipose tissue when cows were fed a higher-energy diet. Data indicated that subcutaneous adipose tissue gene transcription responds more strongly to level of dietary energy, e.g. changes in mRNA abundance encompass not only metabolic pathways but also those involved in the regulation of immune function and inflammation. Although increased secretion of pro-inflammatory cytokines (e.g., TNFα, IL-1β, IL-6) due to obesity is a well-known mechanism associated with systemic chronic low-grade inflammation, reduced appetite, fatty liver disease, and insulin resistance in non-ruminants [52, 53], we did not detect differences in abundance of pro-inflammatory cytokines (*TNF*, *IL1B* and *IL6*) between dietary treatments. On the contrary, the upstream regulators analysis revealed an inhibition of *TNF* before parturition in OVR cows, which had a greater increase in BCS during the dry period [12].

The present findings of a weak pro-inflammatory response in adipose to higher-energy feeding are supported by a recent *in vitro* study by Lopreiato et al. [38] who challenged subcutaneous adipose tissue with bovine recombinant TNF. Furthermore, that study linked the response to greater expression of *PPARG* which in non-ruminants plays a fundamental regulatory role in the attenuation and counter regulation of inflammatory phenomena in adipose tissue. Based on *in vitro* data, activation of PPARγ can attenuate the negative metabolic effects of TNF-α on adipocytes, preventing a decrease in insulin-mediated glucose uptake [54]. It is unclear what role (if any) the localized adipose inflammatory response may exert over the genesis of inflammatory conditions typical of the transition period, especially after parturition, in dairy cows [55, 56].

### Summary and conclusions

**Fig. 7:**
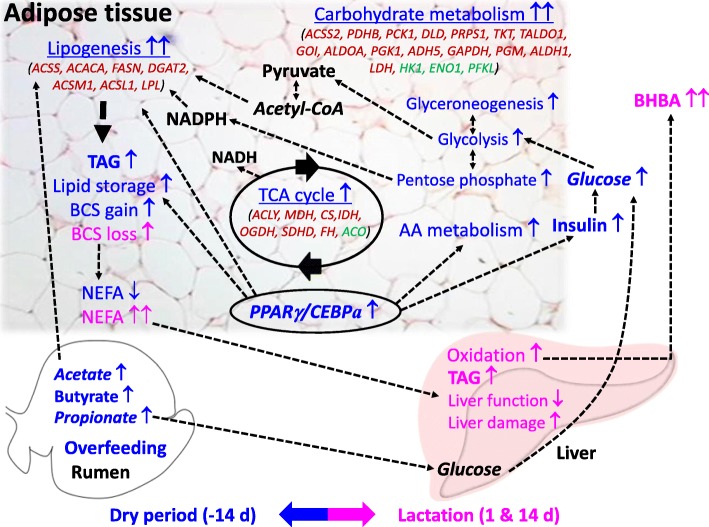
All-encompassing theoretical model of the effect of overfeeding energy during the dry period. Information reported in the model is based on the main findings from the present study and Janovick et al. [15] that highlight key differences between cows overfed (OVR) compared with cows underfed (CTR) energy prepartum. The model includes information relative to adipose tissue gene expression, plasma metabolic parameters, selected aspects of liver metabolism and some assumptions pertaining to ruminal fermentation. Information in blue indicates events occurring in the dry period (− 14 d), while information in pink indicates events occurring during lactation (1 and 14 d). Differentially expressed genes in the main KEGG categories are reported as gene symbols, with red and green color indicating up-regulation or down-regulation, respectively. Down arrows (↓) or up arrows (↑) denote a reduction or inhibition of the respective items. The dotted arrows indicate a link between items

Overfeeding energy during the dry period channels large amounts of energy substrates (e.g. acetate and propionate) to the adipose tissue for storage as triacylglycerol. The transcriptomic approach allowed us to uncover that the adipose tissue responds rapidly to overfeeding mainly under mechanisms of transcriptional control through activation of genes involved in lipid accumulation and amino acid metabolism. In this respect, the nuclear receptor PPARγ acts as one of the main players controlling transcription of genes involved in lipogenesis, but also coordinates catabolism of amino acids that likely become essential for promoting adipocyte differentiation and lipogenesis. Instead, despite energy overfeeding during the dry period, the early postpartum period is characterized by a complete loss of lipogenic gene transcription, where lipolysis appears mainly controlled through other mechanisms, e.g., post-translational activation by protein phosphorylation. As a physiological consequence, the carryover effect of overfeeding energy prepartum affects metabolism drastically, leading to higher NEFA and BHB levels coupled with greater degree of BCS loss (Fig. 7). However, these results underscore the fact that the “detrimental” effects of prepartum overfeeding (discerned from plasma concentrations of energy balance biomarkers) does not necessarily correlate with transcriptional changes in adipose. Lastly, these results underscore the key role of a controlled plan of energy nutrition prepartum, in particular avoiding excess intake of nonstructural carbohydrates, which in turn could increase the likelihood of metabolic disorders in the early post-partum.

## Supplementary information

**Additional file 1.** Complete database of differentially expressed genes (DEG) of bovine subcutaneous adipose tissue from end of pregnancy (− 14 d) through early lactation (1 and 14 d) in dairy cow fed a control diet prepartum (CTR) or relatively high energy diet prepartum (OVR).

**Additional file 2.** Complete result datasets generated by the DIA analysis from transcript profiling analysis in bovine subcutaneous adipose tissue from end of pregnancy (− 14 d) through early lactation (1 and 14 d) in dairy cow fed a control diet prepartum (CTR) or relatively high energy diet prepartum (OVR). The KEGG pathways are sorted by category and subcategory. Blue bars represent the Impact, while red and green bars depict the Direction of the Impact (red = upregulation, green = downregulation)

**Additional file 3.** Complete metabolic and non-metabolic pathways comparison generated by Ingenuity Pathway Analysis from transcript profiling analysis in bovine subcutaneous adipose tissue from end of pregnancy (− 14 d) through early lactation (1 and 14 d) in dairy cow fed a control diet prepartum (CTR) or relatively high energy diet prepartum (OVR).

## Data Availability

The datasets during and/or analyzed during the current study are available from the corresponding author on reasonable request.
